# A Dynamic Multi-Tissue Flux Balance Model Captures Carbon and Nitrogen Metabolism and Optimal Resource Partitioning During Arabidopsis Growth

**DOI:** 10.3389/fpls.2018.00884

**Published:** 2018-06-26

**Authors:** Rahul Shaw, C. Y. Maurice Cheung

**Affiliations:** Division of Science, Yale-NUS College, Singapore, Singapore

**Keywords:** dynamic flux balance analysis, genome-scale metabolic modeling, seedling growth, resource allocation, multi-tissue model

## Abstract

Plant metabolism is highly adapted in response to its surrounding for acquiring limiting resources. In this study, a dynamic flux balance modeling framework with a multi-tissue (leaf and root) diel genome-scale metabolic model of *Arabidopsis thaliana* was developed and applied to investigate the reprogramming of plant metabolism through multiple growth stages under different nutrient availability. The framework allowed the modeling of optimal partitioning of resources and biomass in leaf and root over diel phases. A qualitative flux map of carbon and nitrogen metabolism was identified which was consistent across growth phases under both nitrogen rich and limiting conditions. Results from the model simulations suggested distinct metabolic roles in nitrogen metabolism played by enzymes with different cofactor specificities. Moreover, the dynamic model was used to predict the effect of physiological or environmental perturbation on the growth of Arabidopsis leaves and roots.

## Introduction

In recent years, genome-scale metabolic models, containing reactions catalyzed by enzymes encoded in an organism's genome, have been an emerging tool to study plant metabolic systems (Sweetlove and Ratcliffe, 2011; Seaver et al., 2012; de Oliveira Dal'Molin and Nielsen, 2013). Genome-scale metabolic models are mathematical presentations of metabolism systems and they can be analyzed using computational approaches such as flux balance analysis (FBA) (Orth et al., 2010). This allows researchers to computationally explore possible and optimal behavior of plant metabolism (Yang and Midmore, 2005; Grafahrend-Belau et al., 2013; Poolman et al., 2013; Cheung et al., 2014; de Oliveira Dal'Molin et al., 2015; Feller et al., 2015; Weraduwage et al., 2015). New biological insights about plants were gained using single cell metabolic models (Poolman et al., 2009, 2013; de Oliveira Dal'Molin et al., 2010a; Cheung et al., 2013). The first large scale model of plant was published in 2009 on barely seed metabolism (Grafahrend-Belau et al., 2009). The model predicted responses in oxygen depletion and enzyme deletion conditions. In addition, the predicted growth rates in different conditions were in accordance with published experimental results. In the same year, a genome-scale metabolic model of *Arabidopsis thaliana* was constructed based on AraCyc database (Poolman et al., 2009). A series of improvement of this model for biochemical representation were achieved by Cheung et al. including accounting for maintenance costs (Cheung et al., 2013), day-night metabolism (Cheung et al., 2014) and enzymatic costs in FBA (Cheung et al., 2015). Another Arabidopsis genome-scale metabolic model, AraGEM, was constructed based on literature evidences and was tested by simulating literature-based plant metabolic functions (de Oliveira Dal'Molin et al., 2010a). AraGEM provided a platform for building and annotating metabolic models of other species (de Oliveira Dal'Molin et al., 2010b; Saha et al., 2011). In 2010, three C4 genome-scale models were constructed for mesophyll and bundle sheath cells to observe C4 photosynthesis (de Oliveira Dal'Molin et al., 2010b) which predicted the classical C4 biosynthesis pathways and important metabolic interactions between the two cell types. Mintz-Oron et al. (2012) developed a computational pipeline for creating fully compartmentalized tissue-specific Arabidopsis genome-scale models from databases such as AraCyc (Mueller et al., 2003) and KEGG (Kanehisa et al., 2017). The models were validated against computational and experimental data to test their predictive ability. The first genome-scale model of rice (*Oryza sativa*) was published in 2013, and was applied to investigate the metabolic behaviors of a mesophyll cell of an expanding leaf at varying light intensities (Poolman et al., 2013). Some important interactions between chloroplast and mitochondria in different light levels along with role of photorespiration in high light were identified from the model. In the same year, the effect of flood and drought stresses were investigated in another rice model representing two tissue types: germinating seeds and photorespiring leaves (Lakshmanan et al., 2013). However, these models are not developed to study diel metabolism for both leaf and root under dynamic growth periods. More recently, the development in modeling the behavior of multi-cellular organisms, including plants, have been extended to connect multiple tissue-types to study their inter-dependencies (Grafahrend-Belau et al., 2013; de Oliveira Dal'Molin et al., 2015). The better representation of higher organisms with multi-tissue metabolic models with diel phases could lead to significantly improved model predictions and allow the modeling of source to sink relation. Most of the current plant genome-scale metabolic models were built using a top-down approach, where annotations from biochemical databases (such as BioCyc Caspi et al., 2016, KEGG Kanehisa et al., 2017, PMN Schlapfer et al., 2017 etc.) have been curated to obtain a functional network of biochemical reactions for different species, and such building procedures in most cases result in multiple problematic features including stoichiometric inconsistencies (Gevorgyan et al., 2008), dead metabolites, and disconnected sub-networks (Poolman et al., 2006). Most of these features were inherited from the databases the models were constructed from (Poolman et al., 2006), which is especially problematic for plants due to the large sizes of plant genomes. To avoid the limitations pertaining to top-down models, Arnold and Nikoloski (2014) used a bottom-up approach to reconstruct a large scale Arabidopsis model relying solely on species specific annotations without the need of gap-filling algorithms.

As for the algorithm used in modeling, the standard technique of FBA, which only predicts single metabolic state, has been extended to dynamic FBA (dFBA) (Mahadevan et al., 2002; Hanly and Henson, 2011), which allows the modeling of metabolic changes over time. dFBA allows to observe dynamic flux distributions i.e., change of qualitative and quantitative fluxes over time (e.g., growth) which serves a platform to analyse the temporal changes in rates of metabolic reactions, metabolite transport, C-N and growth material partitioning across organs etc. dFBA has been extensively used to study growth of microbial organisms (Mahadevan et al., 2002; Hanly and Henson, 2011) and it has also been applied in plants (Grafahrend-Belau et al., 2013).

Being a sessile organism, plants have evolved ways to adapt to changes in their environments such as the availability of nutrients and light. Such adaptations include reprogramming of metabolism and changes in resource allocation. In this regard, many researchers have considered the balanced growth (or optimal partitioning) theory (Shipley and Meziane, 2002; Berendse and Möller, 2009; Guo et al., 2016) to study resource allocation during growth under different environments. Balanced growth has been supported by studies in many herbaceous plants (Shipley and Meziane, 2002) and observations of inhibitory effect of high rates of nitrate on root growth of individual *Arabidopsis thaliana* (Zhang and Forde, 2000) and growth in individually grown flowering plants with high/low soil nutrients (specifically nitrogen) (Berendse and Möller, 2009; Guo et al., 2016).

The different organs in plants are interdependent and their metabolism is coordinated during their growth (de Oliveira Dal'Molin et al., 2015; Feller et al., 2015). This can be represented by integrating multiple single models to generate a multi-tissue model (Grafahrend-Belau et al., 2013; de Oliveira Dal'Molin et al., 2015). The multi-tissue model can be used with dFBA to simulate the shifts in metabolism in a growing plant over diel cycle. In this study, we have upgraded an existing Arabidopsis genome-scale metabolic model (Cheung et al., 2014) to a multi-tissue model and introduced a novel method to simulate its dynamic changes in metabolism per day basis under different available resources. To represent a significant growth period, we have considered plant growth right after germination to maturity of the rosette, allowing the model to optimize allocation of resources based on nitrate availability and incident photon. The advantage of using diel metabolic model in dFBA is that it allows comprehensive study of plant growth under light and dark phases of different growth stages. The multi-tissue dynamic FBA modeling framework allows the investigation of qualitative and quantitative metabolic reprogramming in plants under different conditions and/or subjected to perturbations. Thus, allocation of C and N can be studied and mechanism of resource partitioning can be analyzed with specific role of some pathways in space and time.

## Materials and methods

### Arabidopsis diel multi-tissue model construction

**Figure 1:**
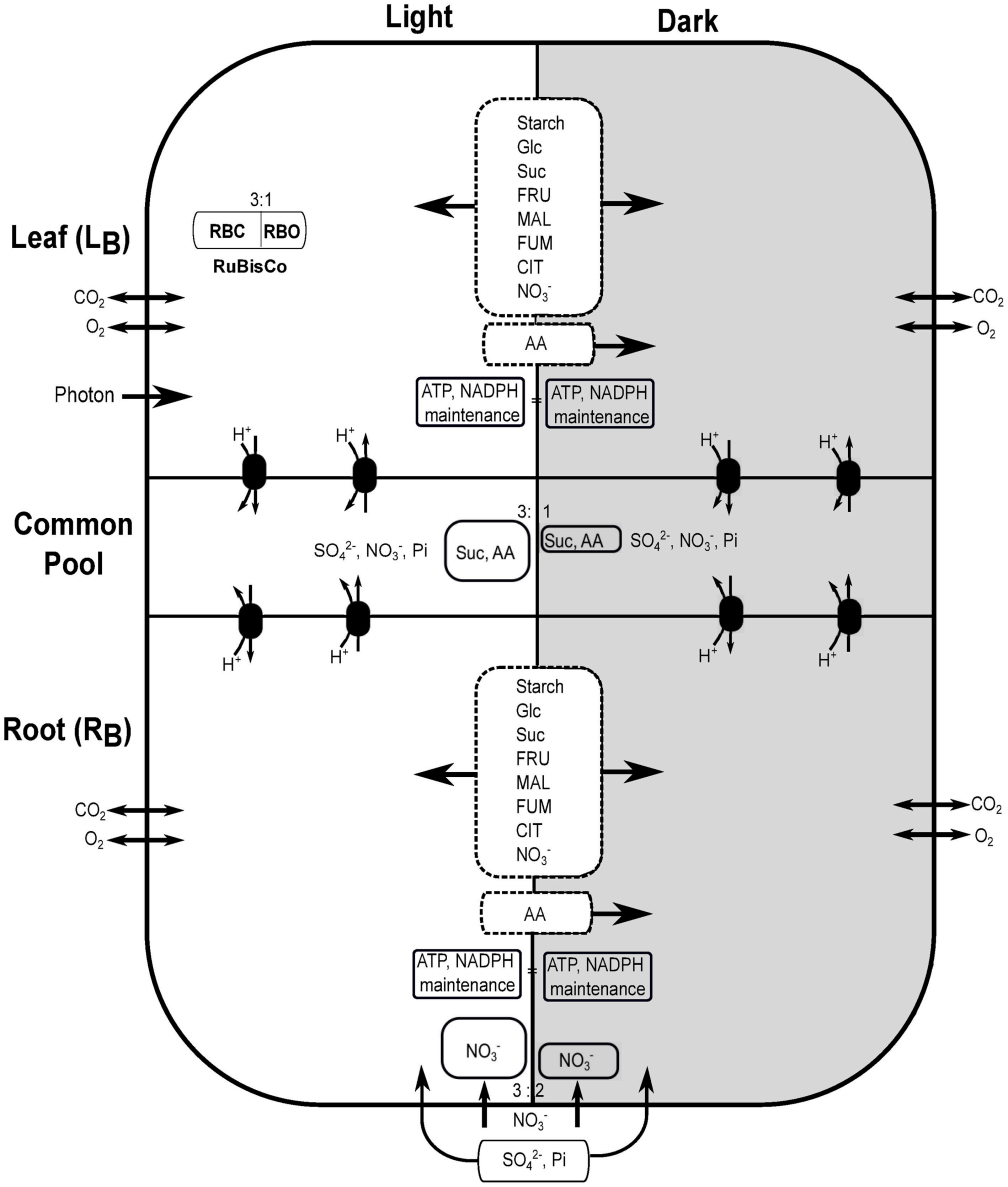
Schematic description of diel leaf-root metabolic model of *Arabidopsis thaliana*. Light and dark phases are represented with white and dark backgrounds, respectively. The ratio of rubisco carboxylase (RBC):oxygenase (RBO) was set to 3:1 in all phases (only leaf in the light is shown). Starch, glucose (Glc), sucrose (Suc), fructose (FRU), malate (MAL), fumarate (FUM), citrate (CIT), and nitrate (${\text{NO}}_{3}^{-}$) were allowed to accumulate in the light and dark phases of leaf and root (dashed rectangle between light and dark where arrows indicate bidirectional storage). AA represents the 18 different amino acids. AA can be stored in the light and utilize in the dark. Exchange of AA, Suc, sulfate (${\text{SO}}_{4}^{2-}$), nitrate and phosphate (Pi) were allowed between leaf and root through the common pool using an active proton pump. Nitrate and photon uptake were allowed through root and leaf, respectively, at a maximum rate quantified by the corresponding tissue biomass. Uptake of other mineral nutrients (${\text{SO}}_{4}^{2-}$, Pi, etc.) were allowed only through the root. Gaseous exchange of CO_2_ and O_2_ were allowed in all four modules.

Genome-scale metabolic model of *Arabidopsis thaliana* (Cheung et al., 2014), previously used to capture the metabolic interactions between day and night, was adapted here to construct a multi-tissue metabolic model with diel phases containing 11,320 reactions (along with exchange reactions) and 10,664 metabolites (Supplementary File 1). Each reaction from the model was duplicated to represent a diel leaf and root model, which created four separated modules, i.e., leaf and root in light and dark phases. The reactions and metabolites of these modules were labeled as, “Leaf_Day,” “Leaf_Night,” “Root_Day,” and “Root_Night,” to represent leaf at light and dark and root at light and dark, respectively. Metabolite transport between root and leaf was implemented by defining a shared resource pool or common pool (CP) representing the phloem (de Oliveira Dal'Molin et al., 2015). Sucrose (Suc), sulfate (${\text{SO}}_{4}^{2-}$), nitrate (${\text{NO}}_{3}^{-}$), phosphate (Pi) and 18 amino acids (as in Cheung et al., 2014) were allowed to be transported between root and leaf through phloem. A number of carbohydrates, organic acids and amino-acids were allowed to accumulate between the light and dark phases of leaf and root as in Cheung et al. (2014). Figure 1 shows the schematic representation of the diel multi-tissue model including metabolite storage and inter-tissue exchanges with their proportional constraints between light and dark phases.

Four individual biomass reactions (Biomass_Leaf/ Root_Day/Night_tx) were used in the respective four modules (light and dark phases of leaf and root), representing the biosynthesis of 39 biomass components, including amino acids, nucleotides, cellulose, sucrose, lignin, fructose, glucose, fatty acid and starch, in observed proportions for leaf and root (Supplementary File 2). The stoichiometric coefficient for the biomass components in leaf and root were obtained from the calculations of de Oliveira Dal'Molin et al. (2015). Light and dark phases in leaf and root have the same biomass component's stoichiometry (i.e., similar synthesizing proportion). These coefficients were normalized to represent one gram biomass (dry weight) production for a unit of flux through the biomass reactions.

### Modeling constraints

Sucrose and amino acid transport between leaf and root during the light and dark phases was constrained at a ratio of 3:1 (light:dark) (Gibon et al., 2004). Amino acid compositions in the phloem were fixed based on the measurements of Arabidopsis phloem (Wilkinson and Douglas, 2003). Nitrate represents the major source of nitrogen (N) in leaf (Macduff and Bakken, 2003) and it is the most common source of N for plants (Tischner, 2000), hence nitrate was set as the sole nitrogen source in the model. Nitrate uptake rate from root was constrained at a ratio of 3:2 under light and dark phases (Delhon et al., 1995; Macduff and Bakken, 2003; Siebrecht et al., 2003). To account for photorespiration, rubisco carboxylase:oxygenase was set at a ratio of 3:1. The reason of these constraints are elaborately described in Cheung et al. (2014). Water and protons in the cytosol, mitochondrion, plastid, peroxisome and vacuole were set as external metabolites. ATP and NADPH requirement for maintenance per gram dry weight (DW) of biomass were estimated to be 7.27 and 2.56 mmol gDW^−1^ day^−1^, respectively, based on the data from Arabidopsis heterotrophic cell suspension culture under control conditions (Supplementary File 3) that accounts a ATP:NADPH ratio of ~3:1 (Cheung et al., 2013). The values of total ATP and NADPH maintenance requirement in a day (24 h) were equally divided in to light (12 h) and dark (12 h) phases (50% each), and the maintenance costs were assumed to be equal for leaf and root per gram of respective tissue biomass. Generic ATPase and NADPH oxidase reactions were used to account for these maintenance costs as used in Cheung et al. (2013). The primary objective function for flux balance analysis was set to maximize whole plant biomass production. The solutions were obtained under the consideration of minimum overall reaction flux (Holzhütter, 2004) that reflects maximum cellular economy to the utilization of enzymatic machinery. To investigate the robustness of the qualitative flux map predicted from the entire growth period using objective functions mentioned before, flux variability analysis (FVA) (Mahadevan and Schilling, 2003) was conducted with the primary objective function of maximizing biomass production and a secondary objective function of minimizing overall reaction flux.

Our approach in modeling resource allocation was based on the balanced growth scheme (Shipley and Meziane, 2002; Berendse and Möller, 2009; Guo et al., 2016). Balanced growth suggests that biomass can be preferentially allocated to leaves or roots so that plants can capture limiting external resources. To implement this scheme in our dFBA growth model which dynamically allocates resources to grow leaf and root based on available resources per day, we have introduced a parameter quantifying the relative growth rates (RGR) of leaf, namely leaf growth proportion (g_r_, see Equation 1), which describes the proportion of overall plant biomass synthesis occurs in the leaf (g_r_) and root (1-g_r_). g_r_ was used to partition leaf (g_r_) and root (1-g_r_) growth proportions (line 11 of Algorithm 1) by encapsulating diminishing (S(t), nitrate) and fixed (P_max_, photon) resources subject to the plant's acquiring capacity, i.e., it estimates the growth proportion of leaf in a day based on the amount of nitrate and photon available under present plant leaf and root biomass that will intake these resources. g_r_ can be modified to simulate growth on multiple limited resources or inclusion of other factors in plant growth in future studies.

(1) $$g_{r}=\frac{\ln(\frac{PLA(t)\;\times P_{\max}}{{}^{\sigma }N_{\max}\;(t)})}{\ln\left(\frac{P_{\max}}{S\left(t\right)}\right)+\;\frac{L_{B}(t)}{R_{B}(t)}}\;=\;\frac{\ln PN_{r}(t)}{\ln\left(\frac{P_{\max}}{S\left(t\right)}\right)+\;\frac{L_{B}(t)}{R_{B}(t)}}$$

Here, PN_r_ represents the ratio of usable photon to possible nitrate uptake. PLA is the projected leaf area, P_max_ is the maximum incident photon (mol photons day^−1^), ^σ^N_max_ (mol gDW^−1^ day^−1^) is the maximum nitrate uptake rate calculated by Michaelis–Menten equation and present root biomass (line 6 of Algorithm 1), S(t) is the available nitrate concentration (mol) and L_B_ and R_B_ are the leaf and root biomass (g), respectively. Michaelis-Menten equation has been used to determine the rate of ion influx to root (Claassen and Barber, 1974) from the external solution given the ion (${\text{NO}}_{3}^{-}$) concentration (S), observed maximum rate of influx (V_max_) and Michaelis constant (K_M_) (the ion concentration where ion influx is one-half of V_max_). Here, we assumed no efflux of ions from root to the external environment as our focus is to study plant growth dynamics driven by absorbed ions rather than root uptake kinetics. Small amount of nitrate (10^−6^ mol) was assumed to be remaining in the last 5 days during maturation to allow feasible solutions with very low growth rates.

### Growth simulation by dynamic flux balance analysis (dFBA)

In this study dFBA was applied to model the dynamic changes in metabolic fluxes over diel cycles during the growth and maturation of Arabidopsis leaves and roots. Algorithm 1 summarizes the steps used for simulating *Arabidopsis thaliana* growth from its seedling to mature vegetative stage i.e., 6–36 days after sowing (DAS) in steps of 1 day (24 h). Michaelis-Menten kinetics was used to determine maximum nitrate uptake rate, ^σ^*N*_*max*_, which depends on the present root biomass. The sum of light phase nitrate uptake rate, ^σ^ *N*_*D*_(*mol gDW*^−1^ *light phase(12 h)*^−1^), and dark phase nitrate uptake rate, ^σ^ *N*_*N*_ (*mol gDW*^−1^ *dark phase(12 h)*^−^^1^), was constrained to be lower or equal to the maximum nitrate uptake rate in a day (^σ^*N*_*max*_, 24 h). Leaf biomass was used to determine the projected leaf area (PLA, m^2^) following the PLA and L_*B*_ data for Arabidopsis given in Weraduwage et al. (2015). Slope of the scatter plot, *m* (m^2^ g^−1^), between PLA and L_*B*_ (Supplementary File 4) was used to scale present leaf biomass (L_*B*_(t)) into corresponding projected leaf area (PLA(t)). Photon influx rate, ^σ^*P* (*mol photons m*^−2^ *light phase(12 h)*^−1^) depends on the maximum irradiance, *P*_*max*_ and present PLA. The objective function (Z) of maximizing whole plant biomass was set by maximizing leaf absolute growth rate (AGR, g day^−1^) at light (^σ^*L*_*D*_) and dark (^σ^*L*_*N*_) along with root AGR at light (^σ^*R*_*D*_) and dark (^σ^*R*_*N*_) phases. Nitrate concentration on each day, S(t), was decreased by the amount nitrate was taken up through the root in a 24 h light-dark cycle. Leaf and root total biomass (L_*B*_ and R_*B*_, respectively) were increased per day in amounts they were produced in 24 h (light + dark).

**Algorithm 1:** Dynamic Flux Balance Analysis (dFBA) for Plant Growth

1: **function** Plant Growth (S(6),L_*B*_ (6), R_*B*_ (6))
2: t = 6
3: **while** Day ≤ 36 **do**
4: *t* ← *t* +1
5: *Day*(*t*) ← *t*
6: ${}^{\sigma }N_{\max}\left(t\right)\leftarrow \;\frac{V_{\max}S(t-1)}{K_{m}+S(t-1)}R_{B}(t-1)$ Michaelis-Menten kinetics
7: ∑ ^σ^N_D_(t),^σ^N_N_(t) ≤ ^σ^N_max_(t)
8: *PLA*(*t*) ← *mL*_*B*_(*t*−1) PLA, Projected Leaf Area
9: ^σ^P(t) ≤ P_max_PLA(t)
10: Maximize Z ← ∑^σ^L_D_,^σ^L_N_,^σ^R_D_,^σ^R_N_
11: ∑ ^σ^*L*_*D*_(t),^σ^*L*_*N*_(t): ∑ ^σ^*R*_*D*_(t),^σ^R_*N*_(t) ← *g*_*r*_(t): (1 –*g*_*r*_(t))
12: *S*(*t*) ← *S*(*t* – 1) – [^σ^*N*_*D*_(*t*)+^σ^*N*_*N*_(*t*)]
13: *L*_*B*_(*t*)←*L*_*B*_(*t*−1) + [^σ^*L*_*D*_(*t*)+^σ^*L*_*N*_(*t*)]
14: *R*_*B*_(*t*)←*R*_*B*_(*t*−1) + [^σ^*R*_*D*_(*t*)+^σ^*R*_*N*_(*t*)]
15: **end while**
16: **end function**

V_*max*_ ~ 0.00336 *mol g DW*^−^^1^ *day*^−^^1^ was used for nitrate transporter in Arabidopsis based on the data from Okamoto et al. (2006) and Gibeaut et al. (1997) (Supplementary File 5). AtNRT1.1 nitrate transporter has two K_M_ values for nitrate and that can vary from 40 μmol to 4 mmol between low and high nitrate concentrations (Parker and Newstead, 2014). In this study, we used the value of K_M_ = 0.4 mmol, slightly less than the maximum saturation value (Krapp et al., 2014) which allowed the model to consume maximum nitrate around mid-growth period (N_low_) and results the best-fit sigmoid growth under our used initial nitrate concentrations.

### Initial parameters

Initial leaf to root biomass ratio was set to 25.8 following the estimates of Weraduwage et al. (2015) in the seedling of Arabidopsis, considering 6 DAS as the start day when cotyledons become fully open (Boyes et al., 2001). 300 μ*mol photons m*^−2^*s*^−1^ was used during the 12 h photoperiod as the maximum incident photon (Havaux and Niyogi, 1999; Varma et al., 1999). In this study, two scenarios with different initial nitrate amount were modeled with 50 mmol and 1.2 mmol used as high (S(6) = N_*high*_) and low (S(6) = N_*low*_) nitrate (Supplementary File 6), respectively at day 6 to commence the growth.

### Software use

Metabolic modeling tool COBRAPy (Ebrahim et al., 2013) was used to implement the method and results were analyzed by the scripts written in Python (www.python.org).

## Results

### Dynamic FBA framework simulated growth dynamics and resource partitioning over multiple developmental stages in arabidopsis

**Figure 2:**
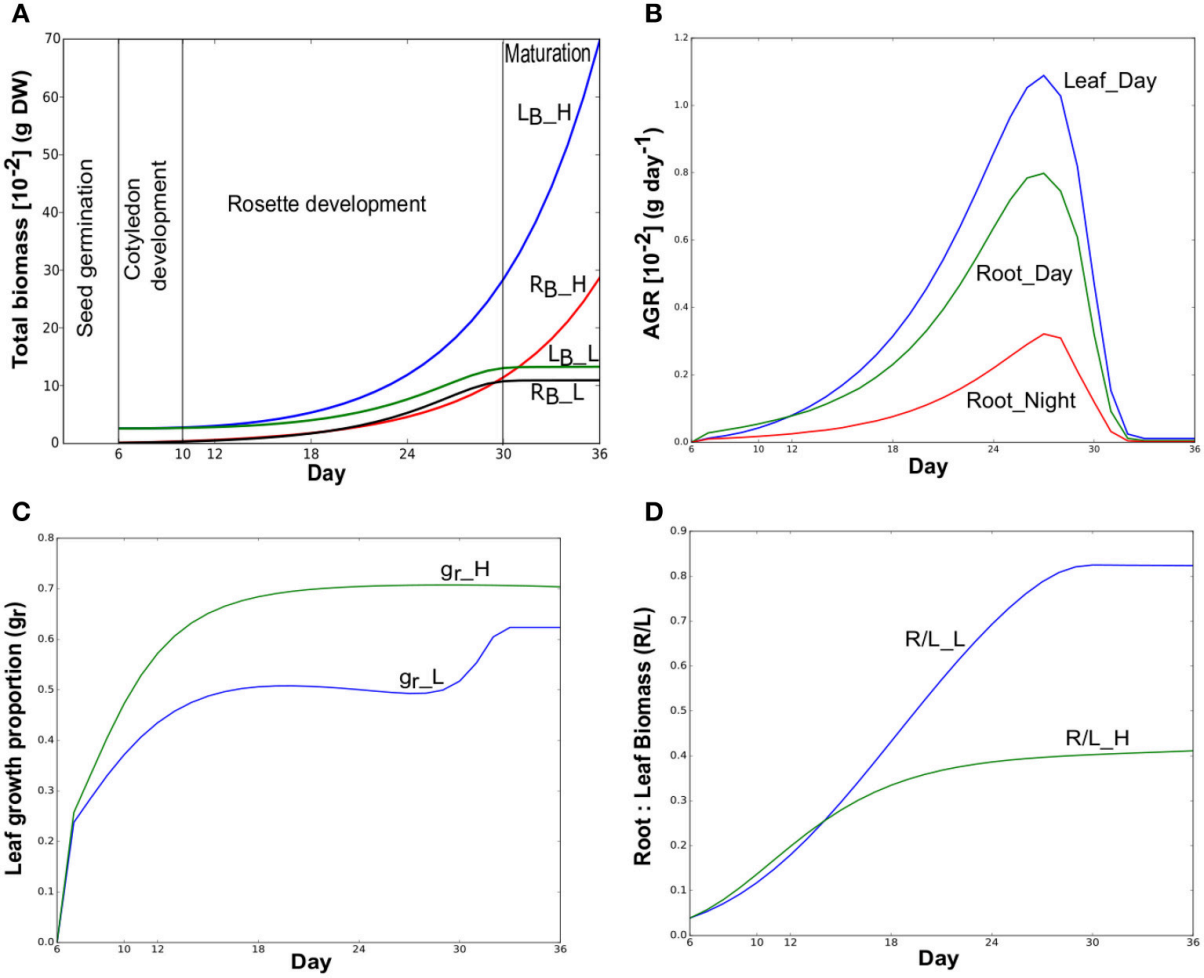
**(A)** Simulated total biomass (dry weight) accumulation of *Arabidopsis thaliana* over time in a 30-day period. Total biomass of leaf (L_*B*_) and root (R_*B*_) under N_*high*_ (_H) and N_*low*_ (_L) are shown here. These suffixes will be used to distinguish reactions in N_*high*_ and N_*low*_ throughout this manuscript. Simulations were started with the cotyledon development (hypocotyl and cotyledon development) stage of Arabidopsis at day 6 that continues upto day 10. Rosette development (leaf and root development) begins from day 11 and completed on day 30 when maturation starts. Days were shown from the day after sowing in all figures. **(B)** Absolute growth rates (AGR) of individual tissues at Day (light) and Night (dark) under N_*low*_. **(C)** Leaf growth proportion (g_*r*_) under N_*high*_ and N_*low*_. **(D)** Root:leaf (R_*B*_:L_*B*_) biomass ratio under N_*high*_ and N_*low*_.

We applied dynamic FBA on a multi-tissue metabolic model of *Arabidopsis thaliana* to explore the metabolic interactions between leaf and root in a diel cycle and the metabolic reprogramming across various growth stages (hypocotyl and cotyledon development, leaf and root development and maturation) under different environment conditions. The dFBA approach predicted the well-known plant growth pattern (Figure 2A) from seedling development to maturation stages. With abundant nitrate (50 mmol), leaf and root total biomass have increased until the day the simulation was ended at day 36. This growth distinctly involves two growth stages (cotyledon development and rosette development), and does not reach maturation since there were no constraints that limit growth under N_*high*_. However, with low nitrate (N_*low*_), diminishing nitrate takes growth into maturation and resulted a sigmoid growth pattern (Yin et al., 2003) of plant growth. This pattern can be considered as the stunted appearance for chronic N deficiency that here begins from the early rosette development stage. In this study, growth under N_*low*_ was analyzed in more detail than N_*high*_ to study the three growth stages including maturation. Figure 2B shows the AGR, i.e, biomass gained per day in leaf and root under N_*low*_ during light and dark phases. Model has predicted leaf growth only in light and root was grown during light and dark phases under both resource conditions.

Growth patterns under both N_*low*_ and N_*high*_ were predicted to first start with more investment in the root as initial root biomass was much smaller than cotyledon leaf (Figure 2C). Under N_*low*_, more growth was partitioned to the root than the leaf (g*r* below 0.5, Figure 2C) from day 6 to day 28. Thus, in N_*low*_, a near-linear increase in R/L during the rosette development occurred (Figure 2D) until day 28. After day 28, there was an increase in leaf growth proportion, but given the lack of available nitrate, the absolute growth rate was so low that it had little impact on the ratio of root to leaf biomass. Figure 2C also shows that leaf growth proportion in N_*high*_ gradually increased and becomes steady until the end of the simulation. In this case, R/L tends toward a value of about 0.4 (Figure 2D) until the end of the simulation as both nutrients (photon and nitrate) were not limiting.

### C:N fixation, quantum demand and assimilation quotient vary with plant development

Carbon fixation through photosynthesis supplies the organic form of carbon molecules such as glucose and sucrose as carbohydrates (Plaxton, 1996; Zheng, 2009). These molecules provide the energy and building blocks for plant growth, maintenance processes and as the food reserve (Stark et al., 1992). Nitrogen uptake and assimilation is a key factor regarding plant growth (Schulze et al., 1994) and gets special attention in crop plants for food (Masclaux-Daubresse et al., 2010). Thus, the ratio of carbon fixation to nitrogen fixation (C_*fix*_/N_*fix*_) and their partitioning is a key determinant for the plant's optimal growth according to nutrient availability (Hermans et al., 2006; Zheng, 2009). Allocation of carbon and nitrogen as growth materials to specific organs effect the size and hence the nutrient capture capacity of the plant. Moreover, the balance of carbon and nitrogen also serves as a signal for up/down regulation of genes for the control of metabolism and cellular responses (Zheng, 2009). Therefore, understanding central metabolism of carbon and nitrogen in plants is a key factor for gaining insight in to plant metabolic behavior and engineering for improved nutrient use efficiency (Sweetlove et al., 2017).

**Figure 3:**
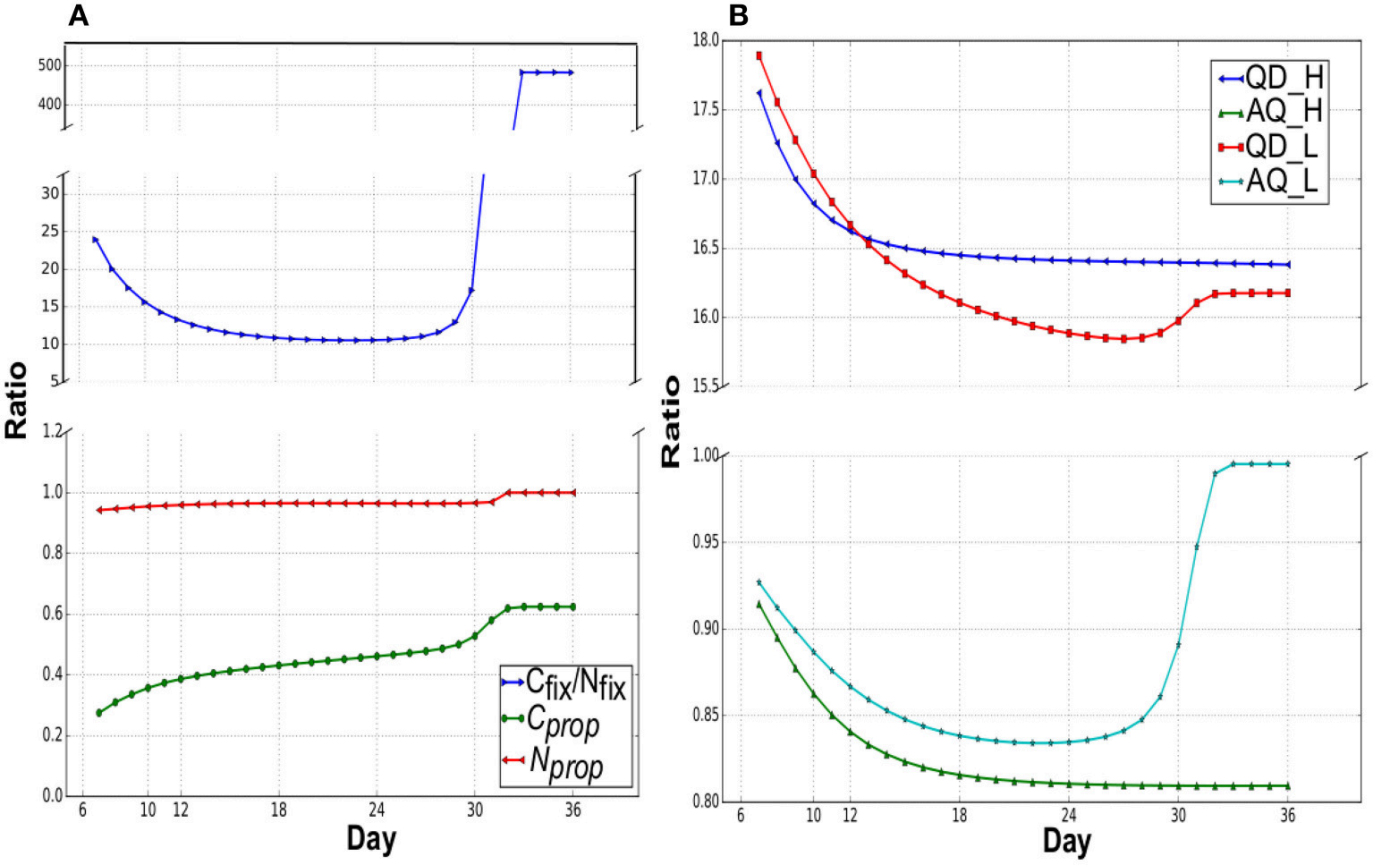
**(A)** Assimilation and partitioning of carbon and nitrogen under N_*low*_. C_*prop*_, and N_*prop*_ are the proportions of C and N allocated to root and leaf, respectively in a day from the total uptake. Remaining C and N were allocated to leaf and root, respectively. C_*fix*_/N_*fix*_ is the ratio of net carbon fixed during the light (flux of CO_2_ transport into leaf during the light phase, CO2_tx_Leaf_Day) to the nitrate uptake in a day (light+dark). **(B)** Quantum demand (QD) and assimilation quotient (AQ) under N_*low*_ and N_*high*_. QD is the ratio of photon required:net CO_2_ fixed (Photon_tx_Leaf_Day:CO2_tx_Leaf_Day i.e., ^σ^P:^σ^CO_2_) and AQ is the net CO_2_ fixed:net O_2_ released (CO2_tx_Leaf_Day:O2_tx_Leaf_Day).

Figure 3A suggests shifting pattern of priorities for C and N partitioning between leaf and root during growth (involving diel cycle) under N_*low*_. C and N partitioned to the different locations were relatively steady under growth in N_*high*_ (data not shown). During the cotyledon development stage, high C_*fix*_/N_*fix*_ indicates that the excess leaf biomass fixes more C than N uptake by small root. There was a gradual decrease in C_*fix*_/N_*fix*_ until day 18 due to the increasing root biomass that can uptake more nitrate. From day 28, just before the maturation phase, there was a sharp increase in C_*fix*_/N_*fix*_ as the nitrate source depletes. During the cotyledon development stage, more fixed carbon was utilized in leaf than transport to root (C_*prop*_ < 0.36). This is mainly due to the small demand for fixed carbon from the root as the initial root biomass was very small. C_*prop*_ gradually increased to above 40% when root biomass increases from exponential growth for more nitrate uptake. During the shift into maturation, proportional increase of fixed carbon partitioning to root was occurred for overcoming very low nitrate concentration. Nitrate was only transported to leaf during the light phases under N_*high*_ and N_*low*_. The proportion of nitrate transported to the leaf from total nitrate uptake (N_*prop*_, Figure 3A) was close to unity in all growth stages, indicating that most imported nitrate was first transported to the leaf during the day.

Quantum Demand (QD) and Assimilation Quotient (AQ) are common measures of efficiency of photosynthesis (John Pirt, 1986; Osborne and Geider, 1987; Cote et al., 1989; Searles and Bloom, 2003; Poolman et al., 2013). QD is defined as the amount of photon required to fix one mole of CO_2_, which is a measure of efficient photon utilization to supply assimilates and energy. AQ is the ratio of net CO_2_ fixed to net O_2_ evolved. Figure 3B shows the change in QD and AQ over different growth stages in N_*low*_ and N_*high*_, which illustrates the relationship between QD and nutrient availability and indicates a continuous metabolic reprogramming in response to change in nutrient availability during plant growth. Maximally efficient QDs were 15.84 and 16.38 in N_*low*_ and N_*high*_, respectively, which was similar to the value of 16.6 calculated from a rice metabolic model (Poolman et al., 2013).

Minimum QD appeared in the mid rosette development stage (day 27) and at the end (day 36) in N_*low*_ and N_*high*_ (Figure 3B), respectively. QD in N_*high*_, steadily decreased to its minimum in the very last day, whereas under N_*low*_, QD has decreased to its minimum at the mid rosette development stage, then increased to a higher value during maturation. The Pearson's correlation coefficients between R/L and QD are −0.8 and −0.89 with a *p*-value of 9 × 10^−8^ and 2.87 × 10^−11^ for N_*low*_ and N_*high*_, respectively, which suggest a negative correlation between root mass growth and QD. It is likely that as root mass increases, there is more N assimilation which leads to a decrease in QD as more energy is diverted from C assimilation to N assimilation. During the start of maturation under N_*low*_, N assimilation started to decrease as external nitrate depletes, which led to a slight increase QD. Similar to QD, the initial decrease in AQ suggests an increase in N assimilation, while an increase in AQ at the maturation phase under N_*low*_ coincides with the decrease in nitrate availability and N assimilation such that AQ approaches 1 as N assimilation approaches 0.

### Enzymes with different cofactor specificities in nitrogen metabolism serve distinct metabolic roles

**Figure 4:**
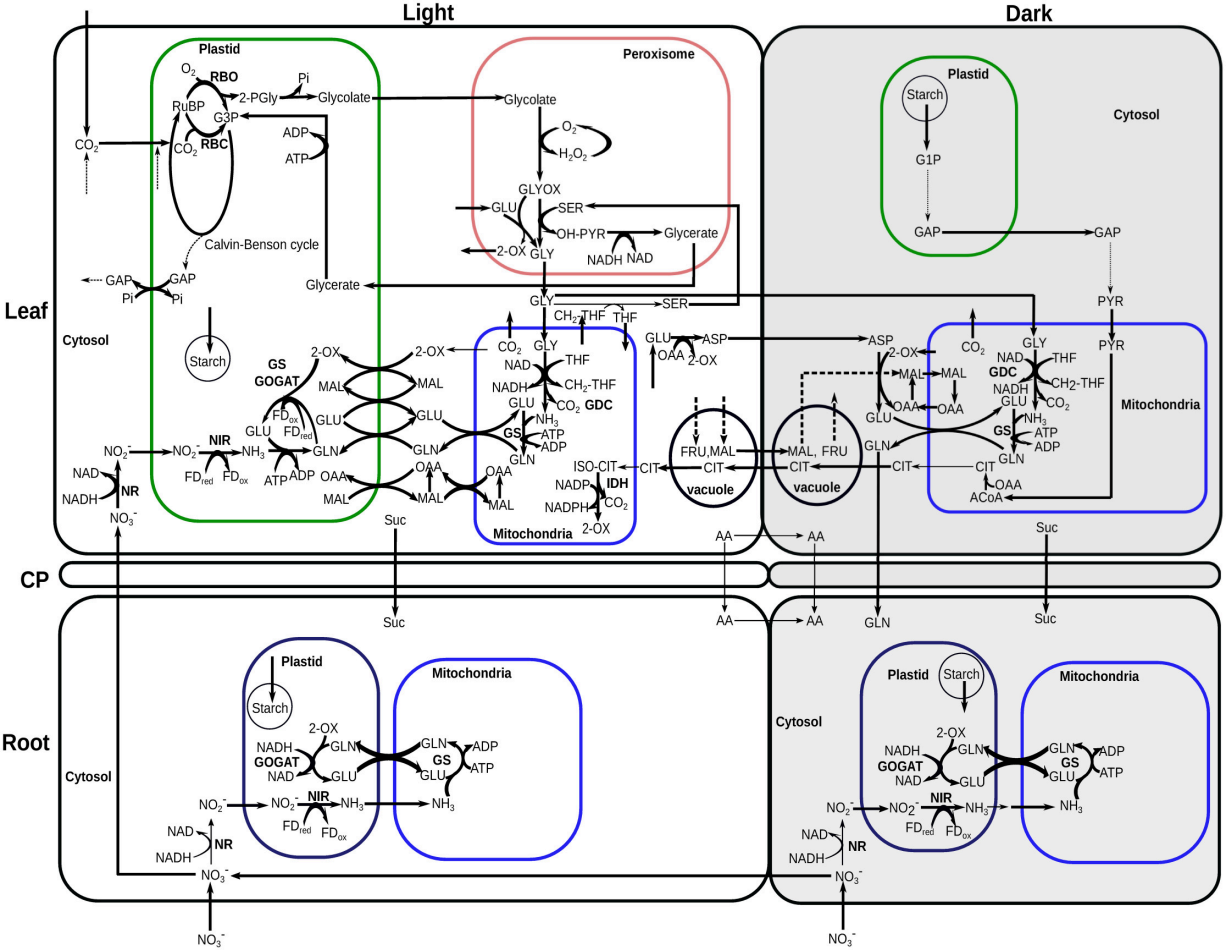
Central carbon and nitrogen metabolism of *Arabidopsis thaliana* active in most growth stages and conditions simulated. Full Calvin-Benson cycle was active in leaf during the light phases. A complete TCA cycle was mainly active at the dark phases of leaf and in light and dark phases of root. Full oxidative pentose phosphate pathway reactions were active at the dark phases in leaf plastid and in light and dark phases of root plastid. These pathways were omitted from the flux map for clarity. The thickness of the arrows is not scaled to relative flux magnitudes. Bold lines indicate reactions identified as essential by FVA under most of the growth days. RuBP, ribulose-1,5-bisphosphate; G3P, 3-phosphoglycerate; 2-PGly, 2-phosphoglycolate; GAP, glyceraldehyde 3-phosphate; Pi, inorganic phosphate; 2-OX, 2-oxoglutarate/α-ketoglutarate; GLU, glutamate; GLN, glutamine; OAA, oxaloacetate; FD_ox_, oxidized-ferredoxins; FD_red_, reduced-ferredoxins; ${\text{NO}}_{2}^{-}$, nitrite; H_2_O_2_, hydrogen-peroxide; GLYOX, glyoxylate; SER, serine; OH-PYR, hydroxypyruvate; GLY, glycine; THF, tetrahydrofolate; CH2-THF, 5,10-methylene-tetrahydrofolate; ASP, aspartate; PYR, pyruvate; G1P, glucose 1-phosphate; ISO-CIT, threo-isocitrate; ACoA, Acetyl-CoA.

Assimilates of C and N are of paramount importance for plant growth and maintenance in all developmental stages. Figure 4 summarizes a qualitative flux map of the central carbon and nitrogen metabolism of a developing Arabidopsis rosette from our multi-tissue diel metabolic model.

From the results of the model simulations, most of the nitrate entered into the root was transported to the leaf, with a small amount of nitrate reduced to nitrite by NADH dependent nitrate reductase (NR) in root cytosol during light and dark phases (Figure 4). The low nitrate reduction in root correlates with the low rate of ammonia assimilation by GS in the root. Nitrate reduction in root was active only until end of the rosette development stage of N_*low*_ and later this stage all of the nitrate was allocated to leaf. This might seem energetically inefficient at first as nitrate has to be transported from the root to the leaf, and assimilated N in the form of amino-acids have to be transported back from the leaf to the root. However, the modeling results suggest that N assimilation in the leaf is more efficient than having to transport more fixed carbon from the leaf to the root to provide the energy for N assimilation in the root.

**Figure 5:**
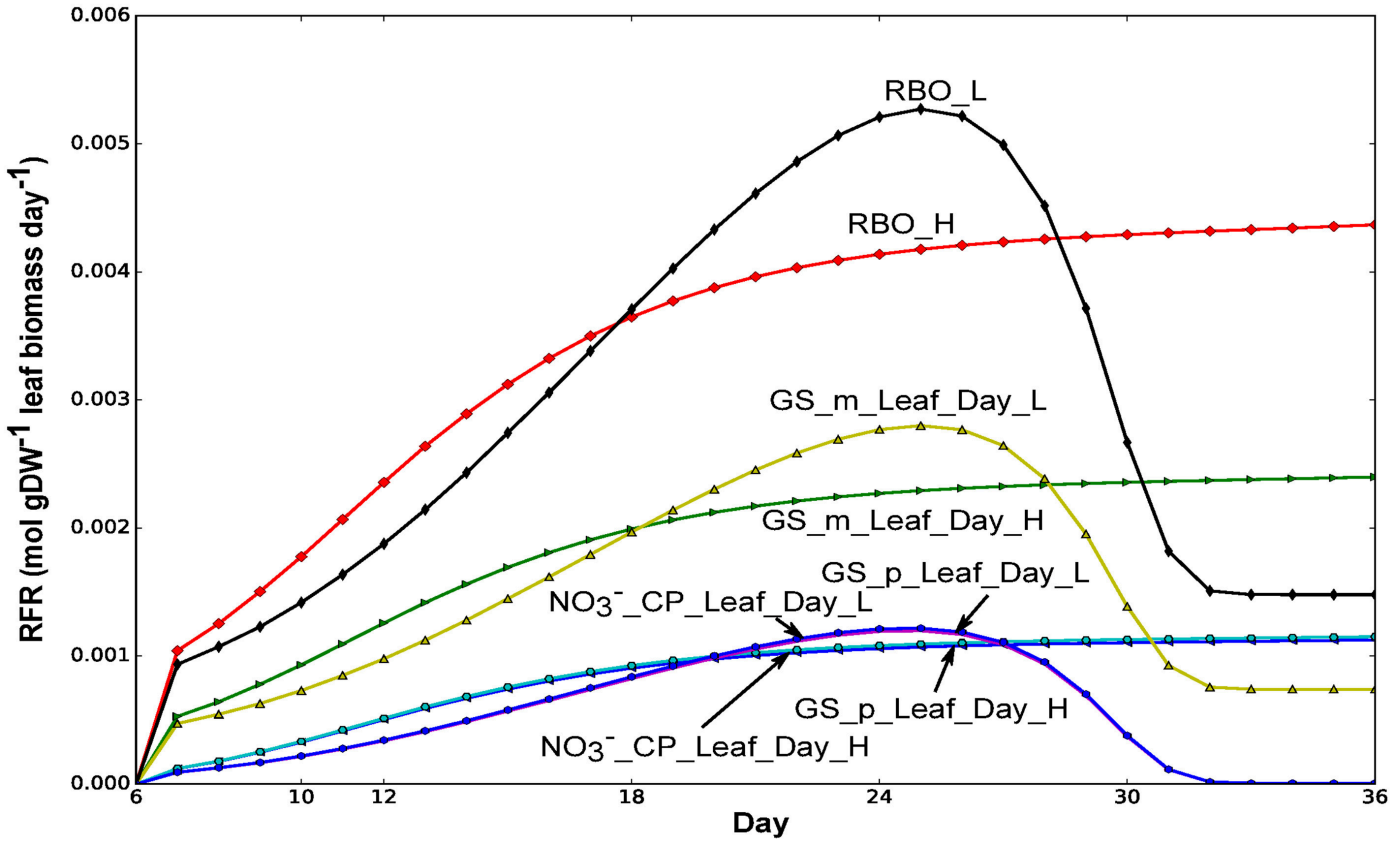
Fluxes of glutamine synthetases (GS) in leaf and nitrate transport from common pool to leaf. Flux values were normalized to per gram leaf biomass. _m and _p represent mitochondrial and plastidic GS, respectively. RFR, relative flux rate.

Under both nitrate concentrations, all of the partitioned N in leaf was first reduced to ammonia (NH_3_) during the light period in plastid by ferredoxin-dependent nitrite reductase (NIR) (Figure 4) then assimilated by plastidic GS (Figure 5). Ammonium assimilation mainly takes place by the combined action of glutamine synthetase (GS) and glutamate synthase (GOGAT) in most plants (Martin-Figueroa et al., 2000; Masclaux-Daubresse et al., 2006). Although cytosolic, mitochondrial and plastidic GS are present in leaf and root, our model predicted only the mitochondrial and plastidial isoforms carried flux (Figures 4, 5). The modeling results suggest that the GS-GOGAT pathway was the main ammonia assimilation pathway which was active in leaf plastid during the light periods in all growth stages using the ferredoxin(Fd)-GOGAT. In root, nitrite was reduced to ammonia by NIR in the plastid, which mostly transported to the mitochondria where it was assimilated by GS to produce glutamine (GLN). GLN was released to the cytosol, and was then imported into the plastid to form glutamate (GLU) by NADH-GOGAT. Experimental study has shown that ammonium assimilation in Arabidopsis root can directly engage NADH-GOGAT, and Fd-GOGAT mainly assimilates ammonium in leaves (Kojima et al., 2014; Konishi et al., 2014). While Fd- and NADH-GOGAT were present both in leaf and root, the model predicted that Fd-GOGAT was the predominant enzyme in the leaf during the day, whereas root primarily uses NADH-GOGAT for ammonia assimilation. This is consistent with experimental results where Fd-GOGATs were found to be around 90% among all GOGATs in shoot (Kojima et al., 2014).

As for GS, our modeling results showed that the plastidial GS flux in the leaf follows that of the nitrate imported in to the leaf during the day in both nitrate conditions tested (Figure 5). This correlation suggests that the net assimilation of N into amino-acids through the GS/GOGAT pathway was primarily carried out by the plastidial GS. In contrast, the mitochondrial GS flux is correlated with the rubisco oxygenase flux, suggesting that the primary function of the mitochondrial GS is the reassimilation of ammonia from the photorespiratory pathway.

At night in the leaf, the stored starch was converted into pyruvate through glycolysis, which was then converted into citrate in the mitochondria via pyruvate decarboxylase and citrate synthase. Some of the citrate was stored at night in vacuole to provide C skeleton in the GS-GOGAT pathway for amino acid biosynthesis during the light phase, which was consistent with the previous experimental and computational studies (Gauthier et al., 2010; Cheung et al., 2014). A recent study on tDT vacuolar malate transporter also showed quantitative changes in citrate levels between light and dark phases under standard conditions (Frei et al., 2018). The maximum daily change in citrate content in our N_low_ simulations at day 25 (at maximum growth rate) is 276.1 μmol/gDW, which is comparable with the experimental measurement of 342.32 μmol/gDW (calculated from 22.0 μmol/gFW, based on a conversion ratio from FW to DW for root tissue) under standard condition. Frei et al. (2018) also observed a reciprocal behavior of citrate levels to the changes in malate under diurnal cycle, which is also apparent in our simulation results, both qualitatively and quantitatively, i.e., there was a similar amount of malate accumulation during the day as compared to the amount of citrate accumulates in the dark (see citrate and malate accumulation in Figure 4). Aspartate and glycine stored during the day in the leaf were used as the main N sources at night to provide substrates for mitochondrial GS for transamination. The resulting glutamine was then released from the leaf to the root.

To confirm the robustness of this qualitative flux map during Arabidopsis growth, we conducted flux variability analysis (FVA) (Mahadevan and Schilling, 2003). Simulated results show that many reactions shown in Figure 4 (bold lines) are essential for most of the growth days to assimilate and partition C-N efficiently. In addition, a sensitivity analysis was performed by varying the following constraints independently, (i) the flux ratio of phloem export between the light and dark phases, (ii) the flux ratio of nitrate uptake between the light and dark phases, (iii) the flux ratio of rubisco carboxylase and oxygenase activity during the light phase, and (iv) the initial nitrate content. Results from the sensitivity analysis confirmed that the pattern of flux distributions shown in Figure 4 was applicable for all scenarios tested (Supplementary File 7).

### Dynamic model predicting responses to physiological and environmental perturbations

**Figure 6:**
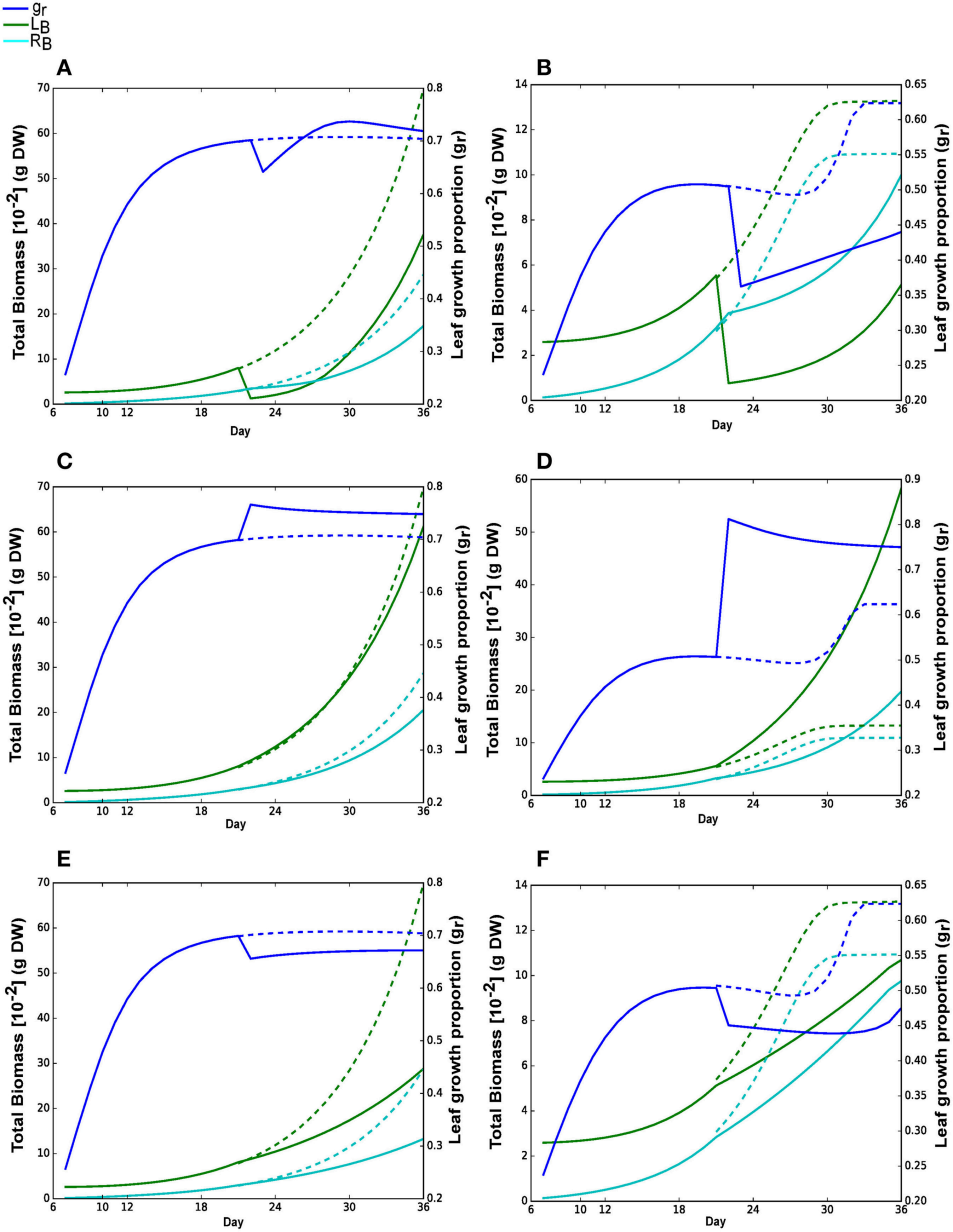
Metabolic responses to physiological and environmental perturbations. All perturbations **(A–F)** were imposed on day 21. Solid lines are the results with perturbations; dashed lines are the growth curves for N_*high*_ or N_*low*_ without perturbation. Blue, green and cyan lines represent leaf growth proportion (g_*r*_), leaf and root biomass, respectively. **(A,B)** Reduction in leaf biomass on day 21 to 0.001 g under N_*high*_ and N_*low*_, respectively. **(C,D)** Increase in nitrate concentration to 100 mmol under N_*high*_ and N_*low*_, where simulations were started with 50 mmol and 1.2 mmol nitrate concentrations under N_*high*_ and N_*low*_, respectively. **(E,F)** Incident photon flux decreased from 300 μ*mol photons m*^−2^ *s*^−1^ to 100 μ*mol photons m*^−2^ *s*^−1^ under N_*high*_ and N_*low*_, respectively.

Figure 6 shows the plant growth performance under different perturbations. A sudden reduction in leaf biomass, simulating the effect of herbivory, resulted in a reduced final leaf and root biomass compared to normal growth (Figures 6A,B). Under high nitrate, leaf biomass was able to recover significantly as sufficient N source was available along with time to complete growth (Figure 6A). Approximately 6 days were required to recover the damage when leaf biomass exceeds the root biomass. However, the recovery was much slower under low nitrate condition and leaf biomass remains smaller than root at the end of the simulation (Figure 6B). Here, most of the photo assimilates were used to recover root growth under low nitrate so that it would capture more nitrate for growth. Increase in nitrate availability, simulating the effect of application of fertilizers, has marginal effect under N_*high*_ as the plant had access to sufficient N source from beginning of the growth (Figure 6C), which is in contrast to a large change in leaf growth proportion under low nitrate growth that resulted in an increase in leaf growth, allowing the plant to fix more carbon per N assimilated (Figure 6D).

A decrease in light intensity (Figures 6E,F), simulating the effect of shades, resulted in a decrease in overall plant growth and a shift in favor of root growth as compared to leaf growth (decrease in leaf growth proportion). This shift in investment could be a response to save resource from growing leaf, and focus the resource on root growth to assimilate more N.

## Discussion

The modeling framework developed in this study provides a tool for predicting the pattern of resource partitioning and the metabolic processes involved across multiple phases of plant development (Supplementary File 8). The method was tested with the genome-scale metabolic model of *Arabidopsis thaliana* in modeling seedling growth to mature rosette leaves. From the day when cotyledon is fully opened, it takes approximately 30 days to mature and initiate flowering (Boyes et al., 2001). The model was able to simulate plant growth stages under different resources as well as abrupt perturbations including the removal of gained biomass by herbivores, addition of nutrients in the soil and shades. To the best of our knowledge, it is the first modeling approach to investigate C, N and growth partitioning in diel phases over multiple developmental stages of *Arabidopsis thaliana* with dynamically imposing environmental and physiological perturbations.

### dFBA modeling framework simulated plant growth dynamics without time-specific growth constraints

Flux balance analysis was first used to represent time profile for the growth of *Escherichia coli* in batch and fed-batch cultures (Varma and Palsson, 1994). This approach was extended incorporating rate of change of flux constraints as dynamic flux balance analysis (dFBA) to observe diauxic growth in *E. coli* (Mahadevan et al., 2002). Since then, dFBA has been applied to model different metabolic systems such as examining microbial growth in substrate mixtures (Hanly and Henson, 2011) and studying life-cycle and synthetic biology prospective for microalgae (Baroukh et al., 2016; Flassig et al., 2016). First multi-tissue model of plant representing leaf-root of barley and their interaction using dFBA was introduced by Grafahrend-Belau et al. (2013), in which the authors used a functional plant model (FPM) to obtain growth kinetics (in time scale) and fed the outputs of FPM to a FBA model as constraints to obtain flux solutions across multiple tissues during seed development from 53 to 70 day after sowing. Our approach differs from Grafahrend-Belau et al. (2013) in that, (i) instead of using separate FPM, the dynamic constraints (present leaf and root biomass, available nitrate and photon, allowed uptake capacity, etc.) were calculated each day, based on the plant growth performance of previous day and available resources (see section Materials and Methods) and (ii) we simulated growth from cotyledon (day 6) to maturation phase (day 36) of Arabidopsis to analyze plant growth under two resource gradients. A recently developed multi-tissue modeling framework using Arabidopsis explores the source-sink interaction and C-N allocation under diel cycle (de Oliveira Dal'Molin et al., 2015). This was the first study to explore the potential of multi-tissue framework in identifying tissue, organ, day period and condition specific pathway correlation. In this study, we extended the scope of such analysis by including growth stages as a time axis, accompanying with a method that can model changes in conditions.

### Growth partitioning for leaf and root is optimized based on nutrient availability for growth

Overall growth pattern shown in Figure 2A showed that the model is capable of simulating the expected plant biomass growth over time (see Figure 3 of Weraduwage et al., 2015). Moreover, the behavior of plants under limiting below ground resources suggests that plant can partition more resources to root to grow it faster than leaf for optimal partitioning (Shipley and Meziane, 2002; Kiba and Krapp, 2016). Zhang and Forde (2000) observed a suppressed lateral root development in 50 mmol KNO_3_ solution, whereas lateral root growth had been observed in 1 mmol KNO_3_ solution in *Arabidopsis thaliana*. This supports the pattern that lateral root elongation can result in higher root biomass under limited N source to acquire more nutrients and compete within the environment. Ericsson (1995) has also observed this pattern with P and S (other major mineral nutrients) along with N. The model predictions were consistent with experiments where the leaf growth proportion is lower (i.e., more resources were allocated to root growth) under low nitrate (Figure 2C). Model also predicted that due to low N allocation in leaf under N_*low*_, its growth gradually decreased starting from day 14 and ultimately end up with significantly lower leaf mass than N_*high*_ (Figure 2A). The ratio of root:leaf (R/L_L) in this case at day 19 (Figure 2D) is similar to experimental value of Arabidopsis root to shoot ratio obtained under ambient CO_2_ and low N media (Hachiya et al., 2014). It was interesting to see that model predicted leaf growth only during photo periods whereas root growth occurs during both light and dark periods under N_*low*_ (Figure 2B). Under our assumption of optimization in FBA, which equates to the efficiencies in the use of available resources and enzymatic machinery for plant growth, the resulting flux solutions showed that the daytime is the more economic phase for leaf growth.

### Photon use efficiency is variable with growth stages and nutrient availability

Modeling results showed that QD is variable across different growth stages depending on the available nutrients and growth partitioning requirements (Figure 3B). This is evident from the QD of the young leaf during cotyledon development stage, which was higher in N_*low*_ than N_*high*_, in contrast to during rosette development stage where QD was lower in N_*low*_ than N_*high*_ as more energy was diverted for N assimilation for leaf growth in N_*high*_ (Figure 3B). This suggests that the photon usage efficiency depends on the necessary C and N assimilation and partitioning for the required leaf:root growth.

### N assimilation preferentially occurs in leaf during daytime

We used our model to investigate the behavior of metabolic reactions involved in nitrogen metabolism during the growth and maturation of Arabidopsis seedling. Figure 5 shows that glutamine synthetase (GS) activity was predicted from our model to be higher both in leaf and root during the light period compared to during the night period, which was consistent with experimental studies in tobacco where the total GS activity was found to be higher during the light period than during the dark period in leaf and root (Matt et al., 2001). In the leaf during the day, the flux of ammonia assimilation by GS in the mitochondrion was predicted to be much higher than that of GS in the plastid (Figure 5), suggesting that there was more photorespiratory ammonia being reassimilated in the mitochondrion compared to ammonia assimilation for net amino-acid synthesis in the plastid. This coincides with experimental studies suggesting that mitochondrial GS activity in light adapted Arabidopsis leaf can be higher than that in the chloroplast as photorespiratory Gly oxidation (Figure 4) can yields large ammonium that needs to be reassimilated (Taira et al., 2004).

All of the nitrogen partitioned to the leaf was assimilated during the light phase in all growth stages in both N_*low*_ and N_*high*_. Besides experimental studies on Arabidopsis (Taira et al., 2004), our modeling results were also in line with the observation in tobacco where illumination increases the activity of nitrate reductase (NR) and high rates of nitrate assimilation can occur during the light period but remain negligible at night, with similar trend also observed for glutamine synthetase activity (Matt et al., 2001; Stitt et al., 2002). Most of the nitrate absorbed into the root in the dark was stored in the vacuole (about 96-99%) and was exported to supply nitrate in leaf during the light phase, whereas nitrate transport from root to leaf was absent during the dark phases. These results suggest that it is energetically preferable to store nitrate during the night which is then transported and fixed in the leaf during the day using the free energy and reducing power directly from the photosynthetic light reaction. Moreover, organic N storage in leaf and its transport to root supports the cycling of nitrogen between root and leaf, and suggests the role of reduced N in phloem sap from source leaf (Dieter Jeschke and Hartung, 2000; Okumoto and Pilot, 2011), presumably to supply nitrogen for root growth.

### Interplays between amino-acids synthesis and related metabolic processes with carbon fixation

**Figure 7:**
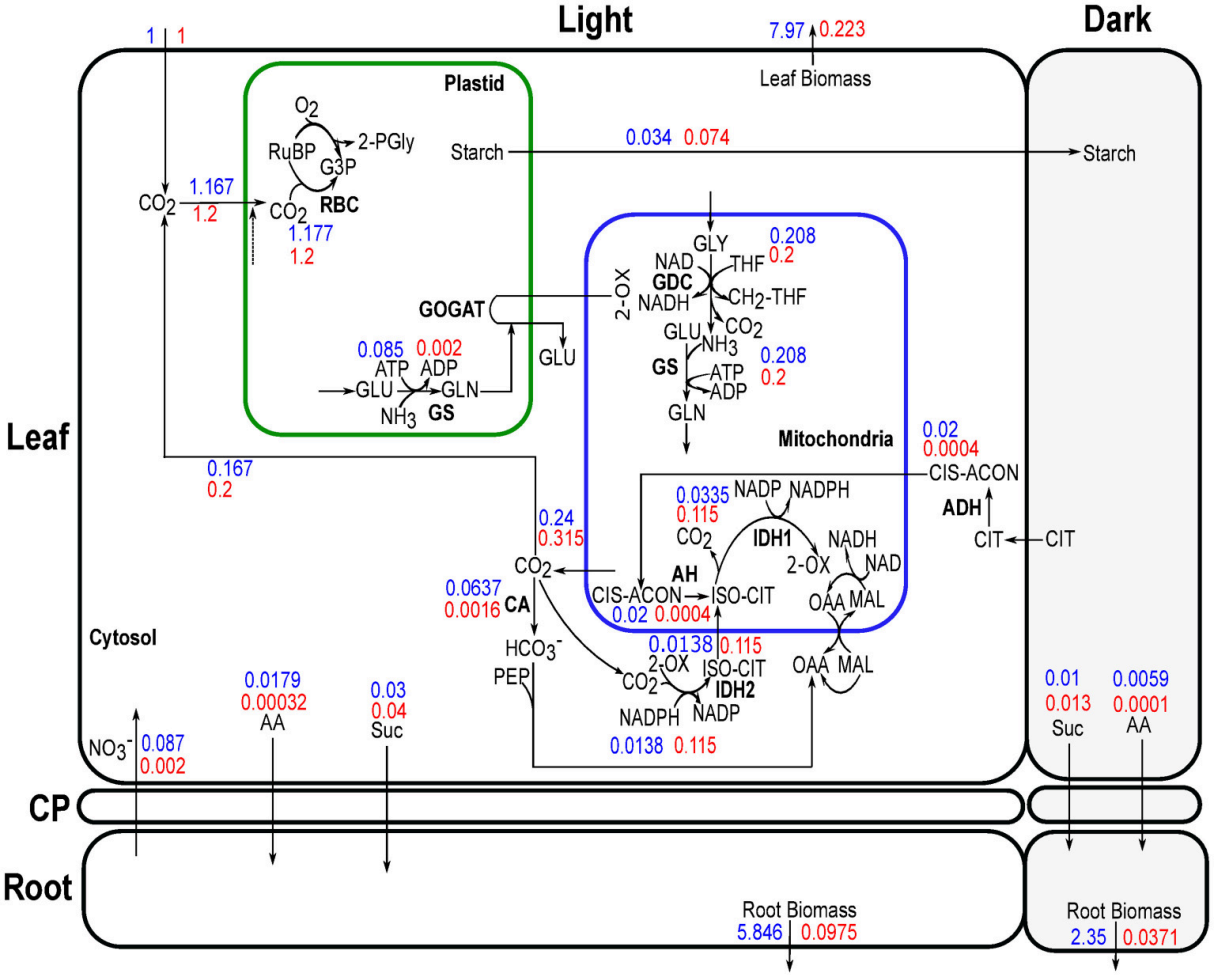
Simulated flux distribution in a day under growing and mature stages of Arabidopsis leaf and root in N_*low*_. The days correspond to lowest QD (15.84) during the rosette development (day 27) and highest QD (16.177) during maturation (day 36) in Figure 3B are shown with blue and red font, respectively. Flux values were normalized to per net CO2 fixed. Flux values for AA represent the normalized sum of all amino acids. Note that the thickness of the arrows is not scaled to relative flux magnitudes. CIS-ACON, cis-aconitate; PEP, phosphoenolpyruvate; ${\text{HCO}}_{3}^{-}$, hydrogen carbonate.

Days corresponding to the lowest and highest QD under N_*low*_ were used to analyze metabolic reprogramming when *Arabidopsis thaliana* growth shifts from rapid rosette development to its maturation under low nitrate condition. Figure 7 shows the metabolic changes between these two stages.

The role of carbonic anhydrase (CA, EC 4.2.1.1) has recently been recognized for amino acid biosynthesis and optimal plant growth in low CO_2_ in Arabidopsis (DiMario et al., 2016). The study with double mutant (β*CAs*) in Arabidopsis leaf suggests that the CAs do not have important link with photosynthesis (DiMario et al., 2016) but has a role in responding to changes in environmental CO_2_ concentrations (Raines et al., 1992). In C4 plants, it has been observed that CA has a significant role to accelerate the rate of photosynthesis (Badger and Price, 1994; Moroney et al., 2001). Our model shows that CA activity can also depend on the C:N assimilation requirement to satisfy metabolic demands. During maturation phase with limited nitrogen source, high C:N assimilation led to low CA activity (Figure 7).

Besides the effect on CA activity, low nitrogen availability could result in lower rates of amino-acids synthesis, which in turn could have a knock on effect on the activities of enzymes involved in providing carbon skeleton for amino-acid synthesis. One example is aconitase (aconitate hydratase, AH) where our model has predicted its localization to be primarily in the mitochondrion under these two stages investigated. It was experimentally observed that reduced expression of AH can enhance rate of photosynthesis in mature leaves of wild-type tomato grown under 250 μ*mol photons m*^−2^*s*^−1^ and available nutrients (Carrari et al., 2003). From our modeling results, it seems that a decrease in AH (or other enzymes involved in the synthesis of carbon skeleton of amino-acids) could lead to a decrease in N fixation, where the energy from the photosynthetic light reactions could be diverted to carbon fixation.

## Conclusion

We developed and applied a multi-tissue dynamic FBA modeling framework with a genome-scale metabolic model of Arabidopsis to study the metabolic reprogramming and changes in resource allocation over the growth and maturation of Arabidopsis seedling under two different initial nutrient levels as well as three different types of perturbations. Our model enables us to investigate metabolic reprogramming that can occur during plant growth over a spatio-temporal resolution, thus, able to study the movement and metabolism of carbon and nitrogen through space, time as well as variations in resource availability and perturbations. Knowledge on the partitioning and metabolism of C and N can guide efforts in metabolic engineering to improve nitrogen use efficiency and photosynthesis.

Our modeling results suggested that plants rely on a common metabolic flux mode during growth and maturation for C and N assimilation and translocation (Figure 4). Primary N fixation directly using the energy from photon was shown to be more economical than the use of catabolic energy based on our modeling prediction that N fixation primarily occurs in the leaf during the day. Our model simulations demonstrated that, based on simple rules of plant-environment and tissue interactions, the whole-plant growth analysis can uncover optimal growth strategies in different stress conditions involving diel phases.

## Author contributions

RS and CYMC performed the model simulations, analyzed the results and co-wrote the manuscript.

### Conflict of interest statement

The authors declare that the research was conducted in the absence of any commercial or financial relationships that could be construed as a potential conflict of interest.

## Supplementary material

Supplementary File 1 Multi-tissue diel metabolic model of *Arabidopsis thaliana* in SBML and spreadsheet formats.

Supplementary File 2 Stoichiometry of the biomass components used in the biomass synthesizing reaction in leaf and root.

Supplementary File 3 Estimates of ATP and NADPH maintenance requirement using cell culture data.

Supplementary File 4 Calculation of leaf biomass (g) to projected leaf area (m^2^) conversion ratio.

Supplementary File 5 Estimate of maximum nitrate uptake rate for Michaelis–Menten equation.

Supplementary File 6 Simulated flux solutions under N_*low*_ and N_*high*_.

Supplementary File 7 Sensitivity analysis by increasing and decreasing day:night constraints and initial nitrate concentration.

Supplementary File 8 Python codes to simulate dynamic plant growth (dFBA) in this study.
