# Supplementary material for: A Dynamic Multi-Tissue Flux Balance Model Captures Carbon and Nitrogen Metabolism and Optimal Resource Partitioning During Arabidopsis Growth
Source: Front Plant Sci. 2018 Jun 26;9:884. doi: 10.3389/fpls.2018.00884 (PMC6028781; doi:10.3389/fpls.2018.00884)
Supplement: Supplementary File 8 — Python codes to simulate dynamic plant growth (dFBA) in this study. [file Data_Sheet_8.ZIP › Supplementary File 8/scobra/scobra_documentation.pdf]

Documentation of scobra

Contents

1 Getting started **3**
- 1.1 Load model . . . 3
- 1.2 Reactions . . . 3
  - 1.2.1 List of reactions . . . 3
  - 1.2.2 Delete reaction(s) . . . 4
  - 1.2.3 Gene reaction association . . . 4
  - 1.2.4 Obtain reaction names ? . . . 5
  - 1.2.5 *Reaction* object . . . 5
    - 1.2.5.1 Reactants and products . . . 6
  - 1.2.6 Number of substances in a reaction - Degree . . . 6
  - 1.2.7 Print reactions . . . 6
  - 1.2.8 Reaction to sub-systems association . . . 7
  - 1.2.9 Add reactions to scobra model . . . 7
  - 1.2.10 Change of reaction stoichiometry . . . 8
- 1.3 Metabolites . . . 8
  - 1.3.1 Get metabolite . . . 9
  - 1.3.2 Get metabolite name . . . 9
  - 1.3.3 Dead metabolites . . . 10
    - 1.3.3.1 Peripheral Metabolites . . . 10
  - 1.3.4 Blocked metabolites . . . 11
  - 1.3.5 Metabolites involved in Reactions - Degree . . . 11
  - 1.3.6 Delete Metabolites . . . 12
  - 1.3.7 Produce Metabolites . . . 12
- 1.4 Genes . . . 12
  - 1.4.1 Get genes . . . 12
  - 1.4.2 Genes to Metabolism . . . 13
  - 1.4.3 Get gene name?? . . . 13
  - 1.4.4 Get gene class object . . . 13
  - 1.4.5 Metabolic link to the genes . . . 13
- 1.5 Reaction and metabolite involvements . . . 14
- 1.6 Neighbours in the metabolic graph . . . 14

2 Flux analysis **16**
- 2.1 Flux balance analysis . . . 16
  - 2.1.1 Set flux bounds to the reactions . . . 17

2.1.2 Set Objective 17
2.1.3 Solving the LP problem 17
2.1.4 Get flux solution 18
2.1.5 Printing the solution 18
2.1.6 Fixing the flux 19
2.1.7 Get sum of fluxes 19
2.1.8 Get Flux bounds 19
2.1.9 Get Overall information of the problem and its solution 20
2.1.10 Get Flux range 20
2.1.11 Get Objective 20
2.1.12 Get status message 21
2.1.13 Get Objective value 21
2.1.14 Get Objective direction 21
2.1.15 Metabolites produced by reactions 21
2.1.16 Degree 21
2.2 flux variability analysis 22

3 Method Implimentation **23**
3.1 MOMA 23
3.1.1 LinearMOMA 23
3.1.2 CleanUpTempVar 23
3.1.3 MOMA2mutant 23
3.2 ROOM 23
3.3 Geometric 23
3.3.1 GeometricSol 23
3.4 Pareto 23

Chapter 1

Getting started

1.1 Load model

Constraints based metabolic modeling tool 'scobra' can handle model files both in sbml and scrumpy formats. These two types of model formats are shown in figure ?? and ??. To load a model, type

m=scobra.Model('PATH-OF-MODEL-FILE')

m = scobra.Model('/home/user/toy.xml') [for SBML model file]<br>m = scobra.Model('/home/user/toy.spy','scrumpy') [for ScrumPy model file]

1.2 Reactions

1.2.1 List of reactions

To obtain a list of reaction ids type, m.Reactions()

```
>>> m.Reactions()
['R1', 'R2', 'R3', 'R4', 'R5', 'R6', 'R7', 'R8']
>>> len(m.Reactions()) [gives count of the reactions]
8
```

1.2.2 Delete reaction(s)

A single reaction can be removed using the command DelReaction. To remove multiple reactions, a list of reactions can be entered as by DelReactions([List of reaction ids])

```
>>> m.DelReaction('R4')
>>> m.Reactions()
['R1', 'R2', 'R3', 'R5', 'R6', 'R7', 'R8']
>>> m.DelReactions(['R1','R5'])
>>> m.Reactions()
['R2', 'R3', 'R6', 'R7', 'R8']
```

1.2.3 Gene reaction association

To obtain reactions/genes associated with genes/reactions, use GenesToReactionsAssociations or ReactionsToGenesAssociations.

```
>>> m.GenesToReactionsAssociations

{'Gr6': ['R6'], 'Gr7': ['R7'], 'Gr4': ['R4'], 'Gr5': ['R5'], 'Gr2': ['R2'], 'Gr3': ['R3'],
'Gr1': ['R1'], 'Gr8': ['R8']}

>>> m.ReactionsToGenesAssociations
{'R4': ['Gr4'], 'R5': ['Gr5'], 'R6': ['Gr6'], 'R7': ['Gr7'], 'R1': ['Gr1'], 'R2': ['Gr2'],
'R3': ['Gr3'], 'R8': ['Gr8']}
```

Here, Gr1, Gr2 etc., are the assumed genes associated with reactions R1, R2 etc., respectively, in the simple toy model.

1.2.4 Obtain reaction names ?

To obtain reaction names one can use the command GetReactionName(Reaction ID).

```
>>> m.GetReactionName('R1')
'R1'
>>> m.GetReactionNames(['R1','R2'])
['R1', 'R2']
```

1.2.5 *Reaction* object

We can get reactions as a *class* object in scobra that can be utilized for several other (useful) built-in *methods*. This can provide important information about the structure/-function of the model. *Reaction* class object can be created as
"r4=m.GetReaction('Reaction ID')" [The reference to the *Reaction* object is assign to 'r4']
Python built-in function 'dir()' can be used to obtain valid methods to be used with r4.

```
>>> r4 = m.GetReaction('R4')
>>> dir(r4)
[list of attributes for the object r4]
>>> r4,r5 = m.GetReactions(['R4','R5']) [to create Reaction objects for multiple reactions]
```

1.2.5.1 Reactants and products

1.2.6 Number of substances in a reaction - Degree

We can obtain a Python dictionary containing keys as the reactions and respective values are the number of involved substances.

```
>>> m.ReactionsDegree()
{'R4': 4, 'R5': 3, 'R6': 2, 'R7': 1, 'R1': 1, 'R2': 1, 'R3': 1, 'R8': 1} [excluding external metabolites/boundaryCondition="true"?]
>>> m.ReactionsDegree()['R5']
3
```

1.2.7 Print reactions

Model reactions can be printed out using the command PrintReactions.

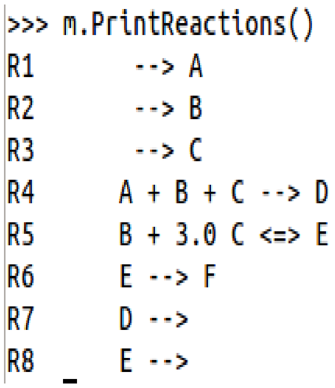
```
>>> m.PrintReactions()
R1       --> A
R2       --> B
R3       --> C
R4      A + B + C --> D
R5      B + 3.0 C <=> E
R6      E --> F
R7      D -->
R8      E -->
```

FIGURE 1.1: All model reactions.

```
>>> m.PrintReaction('R5') [for a single reaction]
R5  B + 3.0 C <=> E
```

1.2.8 Reaction to sub-systems association

We can obtain the part(s) of the metabolism in which the reactions are involved by typing ReactionsToSubsystemsAssociations. This method is applicable to model object created with sbml model format (m). Presently, ScrumPy model format does not supports to provide metabolic localization of a reaction in the model file.???? XR1_Metabolism, XR2_Metabolism etc., are the assumed SUBSYSTEM of reactions R1, R2 etc., respectively.

```
>>> m.ReactionsToSubsystemsAssociations
{'R4': ['XR4_Metabolism'], 'R5': ['XR5_Metabolism'], 'R6': ['XR6_Metabolism'],
'R7': ['XR7_Metabolism'], 'R1': ['XR1_Metabolism'], 'R2': ['XR2_Metabolism'], 'R3':
['XR3_Metabolism'], 'R8': ['XR8_Metabolism']}
>>> m1.ReactionsToSubsystemsAssociations [m1 model object created by ScrumPy
format]
{'R4': [], 'R5': [], 'R6': [], 'R7': [], 'R1': [], 'R2': [], 'R3': [], 'R8': []}
>>> m.SubsystemsToReactionsAssociations [for subsystem to reaction association]
{'XR3_Metabolism': ['R3'], 'XR7_Metabolism': ['R7'], 'XR4_Metabolism': ['R4'],
'XR2_Metabolism': ['R2'], 'XR1_Metabolism': ['R1'], 'XR8_Metabolism': ['R8'],
'XR6_Metabolism': ['R6'], 'XR5_Metabolism': ['R5']}
```

1.2.9 Add reactions to scobra model

Reaction(s) can be added to the scobra model using the method AddReaction. Arguments in the method should be passed to describe a reaction. For example, new reaction 'R9' is added to the model object that includes reactants E and C (stoichiometric coefficient = (negative) 1 and (negative) 2, respectively) and product F (stoichiometric coefficient = (positive) 1). Reversible and irreversible reaction can be described by the third argument.

```
>>> m.AddReaction('R9','E':-1,'C':-2,'F':1)
>>> m.PrintReaction('R9')
R9  2 C + E --> F [irreversible]
>>> m.DelReactions(['R9'])
>>> m.AddReaction('R9','E':-1,'C':-2,'F':1,True)
>>> m.PrintReaction('R9')
R9  2 C + E <=> F [reversible]
```

1.2.10 Change of reaction stoichiometry

Existing reaction's stoichiometry can be changed using ChangeReactionStoichiometry. Just to note that this method can also update a reaction with change in reactants and/or products (with existing set of metabolites).

```
>>> m.ChangeReactionStoichiometry('R9','E':-1,'C':-3,'F':2)
>>> m.PrintReaction('R9')
R9  3 C + E <=> 2 F
>>> m.ChangeReactionStoichiometry('R9','E':-1,'C':3,'F':-2)
>>> m.PrintReaction('R9')
R9  E + 2 F <=> 3 C
>>> m.ChangeReactionStoichiometry('R9','E':-1,'C':3,'F':-2,'A':1)
>>> m.PrintReaction('R9')
R9  E + 2 F <=> A + 3 C
```

1.3 Metabolites

To get a list of metabolites present in the scobra model use the following command.

```
>>> m.Metabolites()
['A', 'B', 'C', 'D', 'E', 'F']
```

1.3.1 Get metabolite

To get metabolite class object(s) use GetMetabolite(Metabolite) or GetMetabolites([list of metabolites]).

```
>>> met=m.GetMetabolite('A')
>>> met.name
'A_internal'
>>> met.reactions [to get the reaction(s) that involve metabolite 'A']
frozenset([<Reaction R1 at 0xa9f8b4c>, <Reaction R4 at 0xa9f8ccc>])
>>> met.compartment
'internal'
>>> met.charge [charge]
-2
>>> metA, MetB = m.GetMetabolites(['A','B']) [for multiple metabolites]
```

1.3.2 Get metabolite name

```
>>> m.GetMetaboliteName('A')
'A'
>>> m.GetMetaboliteNames(['A','B'])
['A', 'B']
```

1.3.3 Dead metabolites

Dead metabolites are those which either produced or consumed by a reaction. Dead end metabolites in the internal stoichiometric model present in the periphery can be identified by DeadEndMetabolites. Removal of a dead end metabolite sometime makes another metabolite as dead, using DeadMetabolites one can find all metabolites those are not involved in any allowed reactions.

```
>>> m.DeadEndMetabolites()
['F']
>>> m.DeadMetabolites()
['F']
>>> m.AddReaction('R9','F':-1,'G':1) [To add a successive dead reaction that utilizes
'F' as a reactant]
>>> m.DeadMetabolites()
['G', 'F']
>>> m.DeadEndMetabolites()
['G']
```

1.3.3.1 Peripheral Metabolites

Instructions are shown with temporary addition of reaction R9.

```
>>> m.PeripheralMetabolites("all")
['F']
>>> m.AddReaction('R9',{'F':1,'G':-1})
>>> m.PrintReactions()
R1     --> A
R2     --> B
R3     --> C
R4    A + B + C --> D
R5    B + 3.0 C <=> E
R6    E --> F
R7    D -->
R8    E -->
R9    G --> F
>>> m.PeripheralMetabolites("all")
['G', 'F']
>>> m.PeripheralMetabolites("Produced")
['F']
>>> m.PeripheralMetabolites("Consumed")
['G']
>>> m.PeripheralMetabolites("Orphan")
['G']
```

FIGURE 1.2: Instructions to obtain information about dead metabolites consumed/produced and orphans.

1.3.4 Blocked metabolites

Metabolites not involved in allowed reactions during flux analysis.

```
>>> m.BlockedMetabolites()
['F']
```

1.3.5 Metabolites involved in Reactions - Degree

```
>>> m.MetabolitesDegree(['B','F'])
{'B': 3, 'F': 1}
>>> m.MetabolitesDegree('B')
{'B': 3}
```

1.3.6 Delete Metabolites

```
>>> m.Metabolites()
['A', 'B', 'C', 'D', 'E', 'F']
>>> m.DelMetabolite('A')
>>> m.Metabolites()
['B', 'C', 'D', 'E', 'F']
>>> m.DelMetabolites(['B','C'])
>>> m.Metabolites()
['D', 'E', 'F']
```

1.3.7 Produce Metabolites

Metabolites those are produced or not can be obtained using ProduceMetabolites. Example is shown using the model given in figure 1.2.

```
>>> m.ProduceMetabolites()
{'Produce': ['A', 'B', 'C', 'D', 'E', 'F'], 'Not Produce': ['G']}
```

1.4 Genes

1.4.1 Get genes

```
>>> m.Genes()
['Gr1', 'Gr2', 'Gr3', 'Gr4', 'Gr5', 'Gr6', 'Gr7', 'Gr8']
```

1.4.2 Genes to Metabolism

```
>>> m.GenesToSubsystemsAssociations
{'Gr6': ['XR6_Metabolism'], 'Gr7': ['XR7_Metabolism'], 'Gr4': ['XR4_Metabolism'],
'Gr5': ['XR5_Metabolism'], 'Gr2': ['XR2_Metabolism'], 'Gr3': ['XR3_Metabolism'],
'Gr1': ['XR1_Metabolism'], 'Gr8': ['XR8_Metabolism']}
```

1.4.3 Get gene name??

```
>>> m.GetGeneName('R1')
'R1'
>>> m.GetGeneName('Gr1')
'Gr1'
>>> m.GetGeneNames(['R1','R2'])
['R1', 'R2']
>>> m.GetGeneNames(['Gr1','Gr2'])
['Gr1', 'Gr2']
```

1.4.4 Get gene class object

```
>>> g1=m.GetGene('Gr1')
>>> g1,g2=m.GetGenes(['Gr1','Gr2'])
```

1.4.5 Metabolic link to the genes

```
>>> m.SubsystemsToGenesAssociations
{'XR3_Metabolism': ['Gr3'], 'XR7_Metabolism': ['Gr7'], 'XR4_Metabolism': ['Gr4'],
```

```
'XR2_Metabolism': ['Gr2'], 'XR1_Metabolism': ['Gr1'], 'XR8_Metabolism': ['Gr8'],
'XR6_Metabolism': ['Gr6'], 'XR5_Metabolism': ['Gr5']}
```

1.5 Reaction and metabolite involvements

```
>>> m.InvolvedWith('A')
{<Reaction R4 at 0xa3ebf4c>: -1.0, <Reaction R1 at 0xa3ebd4c>: 1.0}
>>> m.InvolvedWith('R1')
{<Metabolite A at 0xa3ebacc>: 1.0}
>>> m.InvolvedWith('R5')
{<Metabolite E at 0xa3ebc0c>: 1.0, <Metabolite C at 0xa3ebbac>: -3.0,
<Metabolite B at 0xa3ebb4c>: -1.0}
```

1.6 Neighbours in the metabolic graph

See figure for explanation.

```
>>> m.GetNeighbours('A')
['C', 'B', 'D']
>>> m.GetNeighbours('R1')
['R4']
>>> m.GetNeighbours('R3')
['R4', 'R5']
>>> m.GetNeighbours('E')
['C', 'B', 'F']
>>> m.GetNeighbours('F')
['E']
>>> m.GetNeighboursAsDic('R3')
```

```
{'C': ['R4', 'R5']}
>>> m.GetNeighboursAsDic('F')
{'R6': ['E']}
```

Chapter 2

Flux analysis

2.1 Flux balance analysis

Scobra model can be used to simulate metabolism using flux balance analysis by model.Solve(). Simulation example is shown here using the simple (toy) model given in figure 2.1 which is the graphical representation of the model shown in ??.

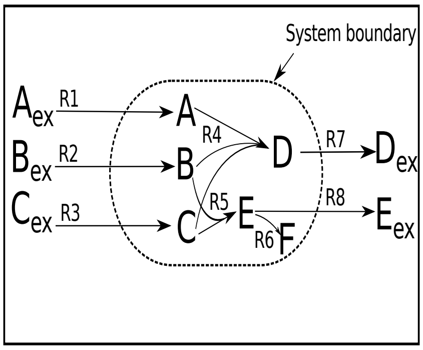

FIGURE 2.1: Simple model

```
m = scobra.Model('/home/user/toy.xml')
```

2.1.1 Set flux bounds to the reactions

Upper and lower limit of a reaction can be set using SetConstraint (for a single reaction) or SetConstraints(dict of reactions and flux bounds).

```
>>> m.SetConstraint('R1',0,10)
>>> m.SetConstraints({'R2':(0,13),'R3':(0,30)})
```

In this example input flux bounds are set for the model shown in figure 2.1.

2.1.2 Set Objective

Here, suppose the objective is to calculate maximum growth rate in R7 and R8, i.e., maximizing Dex and Eex production. The objective reactions and its maximization or minimization can be set using SetObjective and SetObjDirec, respectively.

```
>>> m.SetObjective(['R7','R8'])
>>> m.SetObjDirec("Max")
```

2.1.3 Solving the LP problem

The constraints based LP problem can be solved using Solve(). For this example, we have got optimal growth rate, i.e., a feasible solution. Problems which are not possible to solve, Solve() will give rise 'infeasible'.

```
>>> m.Solve()
optimal
```

2.1.4 Get flux solution

To get the flux solution in dictionary form use GetSol.

```
>>> m.GetSol()
{'R4': 10.0, 'R5': 3.0, 'R7': 10.0, 'R1': 10.0, 'R2': 13.0, 'R3': 19.0, 'R8': 3.0}
>>> from Data import DataSets
>>> ds=DataSets.DataSet()
>>> sol=m.GetSol()
>>> ds.UpdateFromDic(sol)
>>> ds.GetCol('R5')
[3.0]
```

2.1.5 Printing the solution

Flux distributions can also be obtained using PrintSol. For our example, the maximum growth rate (maximum possible flux under the input flux assumptions) for R7 and R8 are 10.0 and 3.0, respectively.

```
>>> m.PrintSol()
R3: 19.0
R2: 13.0
R7: 10.0
R4: 10.0
R1: 10.0
R8: 3.0
R5: 3.0
```

2.1.6 Fixing the flux

Flux through a reaction can be fixed using SetFixedFlux.

```
>>> m.SetFixedFlux('R1':0)
```

2.1.7 Get sum of fluxes

Get the sum of fluxes for a metabolite using FluxSum(Metabolite). Please refer to the figure 1.1 and solution space (section 2.1.5) for this output.

```
>>> m.FluxSum('E')
3.0
>>> m.FluxSum('B')
13.0
>>> m.FluxSum('C')
19.0
```

2.1.8 Get Flux bounds

```
>>> m.GetConstraint('R1')
(0, 10)
>>> m.GetConstraints()
{'R4': (0.0, 1000.0), 'R5': (-1000.0, 1000.0), 'R6': (0.0, 1000.0), 'R7': (0.0, 1000.0),
'R1': (0, 10), 'R2': (0, 13), 'R3': (0, 30), 'R8': (0.0, 1000.0)}
```

2.1.9 Get Overall information of the problem and its solution

```
>>> m.GetState()
{'objective_direction': 'maximize', 'solver': None, 'bounds': 1000.0, 'solution':
<Solution 13.00 at 0xafdc7cc>, 'objective': {'R4': 0.0, 'R5': 0.0, 'R6': 0.0, 'R7': 1,
'R1': 0.0, 'R2': 0.0, 'R3': 0.0, 'R8': 1}, 'quaduatic_component': None, 'constraints':
{'R4': (0.0, 1000.0), 'R5': (-1000.0, 1000.0), 'R6': (0.0, 1000.0), 'R7': (0.0, 1000.0),
'R1': (0, 10), 'R2': (0, 13), 'R3': (0, 30), 'R8': (0.0, 1000.0)}}
```

2.1.10 Get Flux range

```
>>> m.AllFluxRange()
'R4': (0.0, 10.0), 'R5': (0.0, 10.0), 'R6': (0.0, 0.0), 'R7': (0.0, 10.0), 'R1': (0.0, 10.0),
'R2': (0.0, 13.0), 'R3': (0.0, 30.0), 'R8': (0.0, 10.0)
>>> m=scobra.Model('/home/user/toy.xml')
>>> m.AllFluxRange()
{'R4': (0.0, 1000.0), 'R5': (0.0, 333.33), 'R6': (0.0, 0.0), 'R7': (0.0, 1000.0), 'R1':
(0.0, 1000.0), 'R2': (0.0, 1000.0), 'R3': (0.0, 1000.0), 'R8': (0.0, 333.33)}

Note
>>> m.GetConstraints()
{'R4': (0.0, 1000.0), 'R5': (-1000.0, 1000.0), 'R6': (0.0, 1000.0), 'R7': (0.0, 1000.0),
'R1': (0.0, 1000.0), 'R2': (0.0, 1000.0), 'R3': (0.0, 1000.0), 'R8': (0.0, 1000.0)}
```

2.1.11 Get Objective

```
>>> m.GetObjective()
{'R7': 1, 'R8': 1}
```

2.1.12 Get status message

```
>>> m.GetStatusMsg()
'optimal'
```

2.1.13 Get Objective value

```
>>> m.GetObjVal()
13.0
```

2.1.14 Get Objective direction

```
>>> m.GetObjDirec()
'maximize'
```

2.1.15 Metabolites produced by reactions

```
>>> m.ProducedBy('B')
{<Reaction R2 at 0xba08d8c>: 1.0, <Reaction R5 at 0xba08fcc>: -1.0}
```

2.1.16 Degree

```
>>> m.Degree('R5')
3
>>> m.Degree('R4')
4
>>> m.Degree('B')
```

```
3
>>> m.Degree('F')
1
>>> m.DegreeDist()
{1: 1, 2: 2, 3: 3}
```

2.2 flux variability analysis

Chapter 3

Method Implimentation

3.1 MOMA

3.1.1 LinearMOMA

3.1.2 CleanUpTempVar

3.1.3 MOMA2mutant

3.2 ROOM

3.3 Geometric

3.3.1 GeometricSol

3.4 Pareto
